# Prevalence of preoperative anxiety among hospitalized patients in a developing country: a study of associated factors

**DOI:** 10.1186/s13741-023-00336-w

**Published:** 2023-08-24

**Authors:** Ramzi Shawahna, Mohammad Jaber, Iyad Maqboul, Hatim Hijaz, Marah Tebi, Nada Al-Sayed Ahmed, Ziyad Shabello

**Affiliations:** 1 Department of Physiology, Pharmacology and Toxicology, Faculty of Medicine and Health Sciences, An-Najah National University, Nablus, Palestine; 2 Clinical Research Center, An-Najah National University Hospital, Nablus, Palestine; 3 Department of Medicine, Faculty of Medicine and Health Sciences, An-Najah National University, Nablus, Palestine; 4 An-Najah National University Hospital, Nablus, Palestine

**Keywords:** Preoperative anxiety, Anxiety, Fear of anesthesia, Preoperative care, Surgical procedures, Surveys and questionnaires

## Abstract

**Background:**

Preoperative anxiety is a health concern among patients scheduled for surgical interventions. Little is known about the prevalence of preoperative anxiety among patients in different healthcare systems of developing countries. This study was conducted to determine the prevalence of preoperative anxiety among patients undergoing surgery in Palestine. Another objective was to identify the factors associated with preoperative anxiety.

**Methods:**

This study was conducted in a cross-sectional descriptive design. Patients scheduled for surgical interventions were interviewed using an interviewer-administered questionnaire. The questionnaire collected the demographic, clinical, and surgical variables of the patients. The questionnaire also contained the Amsterdam preoperative anxiety and information scale (APAIS), and a short version of the Spielberger state-trait anxiety inventory (STAIS-5/STAIT-5).

**Result:**

A total of 280 patients were included. The mean APAIS total score was 13.6 ± 5.9, the mean APAIS anxiety domain score was 8.3 ± 4.3, and the mean APAIS need for information domain was 1.6 ± 0.50. Of the patients, 76 (27.1%) had high anxiety and 160 (57.1%) expressed a high need for information. The higher APAIS anxiety scores were predicted by being female, having chronic diseases, being scheduled to be operated on within 24 h, and having experienced surgical complications. The mean STAIS-5 score was 10.0 ± 4.2 and the mean STAIT-5 was 10.3 ± 3.8. Of the patients, 140 (50.0%) had high state anxiety and 56 (20.0%) had high trait anxiety. Higher STAIS-5 scores were predicted by being female, younger than 42 years, and scheduled to be operated on within 24 h. Higher STAIT-5 scores were predicted by being female. A positive correlation was identified between APAIS total, APAIS anxiety, APAIS need for information, STAIS-5, and STAIT-5 scores.

**Conclusion:**

Preoperative anxiety was prevalent among patients scheduled for surgical operations in Palestinian hospitals. Anesthesiologists and other providers of perioperative care should screen preoperative patients who are female, have chronic diseases, are scheduled to be operated on within 24 h, and having had experienced surgical complications for preoperative anxiety. More studies are still needed to investigate the effects of the implemented measures on the prevalence of preoperative anxiety.

**Supplementary Information:**

The online version contains supplementary material available at 10.1186/s13741-023-00336-w.

## Background 

Surgical and medical interventions carried out under general, regional, and local anesthesia are common. It has been estimated that annually more than 230 million major surgical interventions are carried out under anesthesia globally (Weiser et al. 2008). Previous studies have reported that the majority of patients undergoing surgery would experience some level of anxiety; therefore, preoperative anxiety has been recognized as a health concern in perioperative care (Eberhart et al. 2020; Abate et al. 2020; Zemła et al. 2019). Anxiety is an unpleasant feeling of dread over something unlikely to happen, such as the feeling of imminent death (Fernández-Donaire et al. 2019). Anxiety is often accompanied by restlessness, fatigue, problems in concentration, and muscular tension. Preoperative anxiety is described as a vague, uneasy feeling the source of which is often nonspecific to the individual (Ayele et al. 2021).

Previous studies have shown that the prevalence and severity of preoperative anxiety were associated with different patient- and healthcare-related factors (Eberhart et al. 2020; Abate et al. 2020; Zemła et al. 2019). Age, gender, marital status, educational level, type of surgery, type of anesthesia, and presence of chronic diseases were reportedly associated with preoperative anxiety (Eberhart et al. 2020; Abate et al. 2020; Zemła et al. 2019; Liu et al. 2022; Moura et al. 2016; Fentie et al. 2022; Bedaso et al. 2022). Of the different factors, worrying about the success of surgery, postoperative course, and complications were the most important factors (Khalili et al. 2019; Mariyam et al. 2020). In a systematic review with meta-analysis that included 14,652 surgical patients, Abate et al. reported that the pooled prevalence rate of preoperative anxiety among the patients was 48% (95% confidence interval (CI) 39 to 47%) (Abate et al. 2020). In another systematic review with meta-analysis that included 5575 surgical patients from 12 low- and middle-income countries, Bedaso et al. reported that the pooled prevalence rate of preoperative anxiety among the patients was 55.7% (95% CI 48.60 to 62.93) (Bedaso et al. 2022).

Anxiety is an unpleasant and disturbing experience that would involve tension, apprehension, uneasiness, and high autonomic activity. Therefore, preoperative anxiety was shown to be associated with significant negative effects on the health and quality of life of the patients. These effects include changes in blood pressure, fluid and electrolyte imbalances, diminished immune responses, and longer wound healing (Bedaso and Ayalew 2019). These negative effects can increase the risk of perioperative complications and delay postoperative recovery. Additionally, preoperative anxiety can increase the intensity of pain, which in turn, would increase the need for higher doses of anesthetics, consumption of analgesics, morbidity, and mortality. Different approaches to reducing or managing preoperative anxiety were described (Kuzminskaitė et al. 2019). These approaches include using anxiolytics, prayers, massage, music therapy, and counseling/conversation with physicians, counselors, or relatives.

Although preoperative anxiety is a common and important health issue in perioperative healthcare, it is often an overlooked health problem in many healthcare systems of developing countries. Currently, little is known about the prevalence of preoperative anxiety among patients undergoing surgery in Palestine and other developing countries. Additionally, little research was done to identify the factors that could be associated with higher or lower levels of preoperative anxiety among patients undergoing surgeries in developing countries. Therefore, this study was conducted to determine the prevalence of preoperative anxiety among patients undergoing surgery in a developing country. Another objective was to identify the factors associated with preoperative anxiety. The findings of this study might have significant implications for the perioperative care of patients being managed in developing countries.

## Methods

### Study design

This study was conducted in a cross-sectional descriptive design to assess the prevalence of preoperative anxiety among hospitalized patients and to identify the factors associated with preoperative anxiety in Palestine as a developing country. The study was conducted and reported in adherence to the strengthening of the reporting of observational studies in epidemiology (STROBE) statement (Vandenbroucke et al. 2007).

### Study population, sample size, and recruitment

The study population was patients scheduled for elective surgeries in different hospitals. The sample size was calculated using an online sample size calculator (www.raosoft.com). The sample size was calculated at a 90% confidence interval (95% CI) tolerating a margin of error of 5%.

In this study, patients scheduled for minor or major elective surgeries were approached and recruited from 14 different hospitals by three final-year medical students (MT, NA, ZS) who were trained to interview patients. The patients were recruited when they met the following inclusion criteria: (1) being older than 18 years and younger than 75 years, (2) being admitted to a surgical ward in a hospital for minor or major elective surgery, (3) willingness to respond to items in a questionnaire, and (4) providing written informed consent. Patients with psychiatric illnesses, cognitive impairments, and those who were admitted for emergency surgeries were not included in this study.

### Study tool

This study was conducted with the help of a data collection form that was developed specifically for this study. The data collection form was based on previous related studies (Eberhart et al. 2020; Abate et al. 2020; Ayele et al. 2021; Moura et al. 2016; Fentie et al. 2022; Bedaso et al. 2022; Khalili et al. 2019; Mariyam et al. 2020; Bedaso and Ayalew 2019; Aust et al. 2018; Gürler et al. 2022; Li et al. 2021; Moerman et al. 1996; Navarro-Gastón and Munuera-Martínez 2020; Yu et al. 2022). The data collection form was organized into sections. The first section collected the demographic, clinical, and surgical variables of the patients including gender, age, marital status, educational level, employment status, place of residence, and household monthly income. Additionally, the patients were asked to self-rate their satisfaction with their social life and religious commitment. The medical and surgical records of the patients were used to collect information on whether the patient had chronic diseases, type of surgery, the timing of surgery, type of anesthesia, type of hospital, whether the patient had previous surgeries, and whether the patient had suffered surgical or anesthesia complications. The second section contained the Amsterdam Preoperative Anxiety and Information Scale (APAIS) (Moerman et al. 1996). This scale contained 6 items on which the patients responded by describing how they felt using a Likert scale of 5-points (1 = not at all, 5 = extremely). The third section contained the short form of the Spielberger State-Trait Anxiety Inventory (STAIS-5/STAIT-5) (Zsido et al. 2020). The scale was of 2 domains (state = 5 items and trait = 5 items, total = 10 items) on which the patients responded using a Likert scale of 1–4 (1 = not at all, 4 = very much). State anxiety described how the patient felt at the moment and trait anxiety described how the patient generally felt.

### Validity and reliability

A pilot study was conducted among 25 patients who did not participate in the larger study. The patients were asked to respond to the questionnaire twice. The test–retest method was used to ensure the reliability and stability of the scores over a short period. The internal consistency (item-relatedness) was investigated using Cronbach’s alpha. In this study, Pearson’s correlation coefficient (Pearson’s* r*) was 0.93 which indicated excellent stability of responses. The Cronbach’s alpha of the 6-item APAIS was 0.77 and the Cronbach’s alpha of the 10 STAIS-5/STAIT-5 items was 0.84 (the Cronbach’s alpha of the 5-item STAIS-5 was 0.86 and the Cronbach’s alpha of the 5 item STAIT-5 was 0.75). These high values indicated that the questionnaire was reliable and internally consistent.

### Data analysis

The data collected in this study were tabulated in Microsoft Excel Spreadsheets. For each patient, the total APAIS, STAIS-5, and STAIT-5 scores were calculated by summing the responses of the patient on each item in the respective scale (Moerman et al. 1996; Zsido et al. 2020). The APAIS anxiety scores were calculated by summing the responses of the patients on items 1, 2, 4, and 5 and the APAIS need for information scores were calculated by summing the responses of the patients on items 3 and 6. A total APAIS anxiety score of < 11 indicated no or minimal anxiety and a total APAIS anxiety score of ≥ 11 indicated high anxiety. A total APAIS need for information score between 2 and 5 indicated no or little need for information and a total APAIS need for information score of > 5 indicated a high need for information. A total STAIS-5 score of > 9.5 indicated high state anxiety and a total STAIT-5 score of > 13.5 indicated high trait anxiety.

The data were entered into IBM SPSS v.21.0. The data were tested for normal distribution using absolute skewness and kurtosis values. The data were considered normally distributed when the absolute skewness values were between − 2 to + 2 and the absolute kurtosis values were between − 7 to + 7. Because the data were normally distributed, means with their respective standard deviation (SD) were used. Scores were compared using t-tests or analysis of variance (ANOVA) as appropriate. To control potentially confounding factors, the variables that were significantly associated in the *t* tests and ANOVA were included in multiple linear regression models. Goodness-of-fit was assessed using statistically significant *R*^2^. Tolerance and variance inflation factor (VIF) values were used to detect multicollinearity issues. Categorical data were compared using Chi-square or Fisher’s exact test (FET) as appropriate. To control potentially confounding factors, the variables that were significantly associated in the chi-square or FET were included in multivariate logistic regression models. Odds ratios (OR) with their 95% confidence intervals (95% CI) were calculated. In this study, statistical significance was indicated by a *p* value of < 0.05. Scores were correlated using Pearson’s correlations. Pearson’s correlation coefficient (Pearson’s *r*) of ≥ 0.70 was arbitrarily considered strong, 0.70 < Pearson’s *r* ≥ 0.30 was considered moderate, and < 0.30 was considered weak.

### Ethical considerations

This study was conducted in compliance with the international ethical standards in the Declaration of Helsinki. The study received approval from the Institutional Review Board (IRB) of An-Najah National University (IRB protocol approval # Nov.3.2021). Approval was also obtained from the Office of Health Education of the Palestinian Ministry of Health. Written informed consent was obtained from each patient. The data collected were treated as confidential and were coded before analysis.

## Results 

The data were collected from 280 patients. The mean age was 42.3 ± 14.7 years. The majority of the patients (61.8%) were scheduled for surgeries in governmental hospitals. Of the patients, 152 (54.3%) were female, 142 (50.7%) were less than 42 years old, 73 (26.1%) were single, 83 (29.6%) had a university education, 138 (49.3%) were currently employed, and 166 (59.3%) lived in urban areas. The majority of the patients reported moderate satisfaction with their household income, social life, and religious commitment. Of the patients, 175 (62.5%) had chronic diseases, 206 (73.6%) were scheduled to be operated on within 24 h, 239 (85.4%) were scheduled to receive general or regional anesthesia, 194 (69.3%) have had previous surgeries, 27 (9.6%) have had surgical complications, and 84 (30.0%) were scheduled to receive general surgery. The detailed demographic, clinical, and surgical variables of the patients are shown in Table 1.

**Table 1:** Detailed demographic, clinical, and surgical variables of the patients (*n* = *280*)

Variable	*n*	%
Gender		
Male	128	45.7
Female	152	54.3
Age (years)		
< 42	142	50.7
≥ 42	138	49.3
Marital status		
Single (never married)	73	26.1
Was married (currently married/divorced/widowed)	207	73.9
Educational level		
School	197	70.4
University	83	29.6
Employment status		
Unemployed	142	50.7
Employed	138	49.3
Place of residence		
Rural	114	40.7
Urban	166	59.3
Self-rated satisfaction with household income		
Low	34	12.1
Moderate	234	83.6
High	12	4.3
Self-rated satisfaction with social life		
Low	15	5.4
Moderate	155	55.4
High	110	39.3
Self-rated satisfaction with religious commitment		
Low	10	3.6
Moderate	156	55.7
High	114	40.7
Presence of chronic disease		
No	105	37.5
Yes	175	62.5
Timing of the scheduled surgery		
Within ≤ 24 h	206	73.6
> 24 h	74	26.4
Type of anesthesia to be used in the scheduled surgery		
General/regional anesthesia	239	85.4
Local anesthesia	41	14.6
Hospital where the surgery will be performed		
Governmental	173	61.8
Private	107	38.2
Have had previous surgery		
No	86	30.7
Yes	194	69.3
Have had surgical complications		
No	253	90.4
Yes	27	9.6
Type of surgery		
General	84	30.0
Obstetrics and gynecology	63	22.5
Orthopedic	44	15.7
Ear, nose, and throat	22	7.9
Urology	25	8.9
Ophthalmology	4	1.4
Neurosurgery	15	5.4
Cardiac surgery/intervention	15	5.4
Minor surgeries/interventions	8	2.9

### APAIS scores

The mean APAIS total score was 13.6 ± 5.9, the mean APAIS anxiety domain score was 8.3 ± 4.3, and the mean APAIS need for information domain was 1.6 ± 0.50. Of the patients, 76 (27.1%) had high anxiety and 160 (57.1%) expressed a high need for information. The detailed responses of the patients are shown in Table 2.

**Table 2:** Detailed responses of the patients on the APAIS items

		Not at all		Little		Moderately		To a high degree		Extremely	
#	Item	*n*	%	*n*	%	*n*	%	*n*	%	*n*	%
1	I am worried about the anesthetic	150	53.6	57	20.4	38	13.6	8	2.9	27	9.6
2	The anesthetic is on my mind continually	194	69.3	35	12.5	20	7.1	9	3.2	22	7.9
3	I would like to know as much as possible about the anesthetic	123	43.9	43	15.4	43	15.4	30	10.7	41	14.6
4	I am worried about the procedure	115	41.1	60	21.4	47	16.8	19	6.8	39	13.9
5	The procedure is on my mind continually	120	42.9	54	19.3	30	10.7	23	8.2	53	18.9
6	I would like to know as much as possible about the procedure	95	33.9	31	11.1	35	12.5	36	12.9	83	29.6

### Association between APAIS scores with demographic, clinical, and surgical variables of the patients 

The *t* tests, ANOVA, chi-square, and Fisher’s exact tests showed that APAIS total and APAIS anxiety scores were significantly higher for the patients who were female, younger than 42 years, married, employed, had chronic diseases, scheduled to be operated on within 24 h, scheduled to receive general or regional anesthesia, had experienced surgical complications, and were scheduled to receive either obstetrical/gynecological, ophthalmological, general, or ear, nose, and throat operation. On the other hand, the APAIS need for information scores were significantly higher for patients who were female, scheduled to be operated on within 24 h, scheduled to receive general or regional anesthesia, whose surgery would be performed in a governmental hospital, and who were scheduled to receive ophthalmological operation. Differences in APAIS scores are shown in Supplementary Table S1 and associations between demographic, clinical, and surgical variables of the patients with anxiety and need for information categories are shown in Supplementary Table S2.

To control potentially confounding factors, the variables that were significantly associated in the *t* tests and ANOVA were included in multiple linear regression models. The models showed that higher APAIS total scores were predicted by being female, scheduled to be operated on within 24 h, and having experienced surgical complications. The higher APAIS anxiety scores were predicted by being female, having chronic diseases, being scheduled to be operated on within 24 h, and having experienced surgical complications. On the other hand, higher APAIS need for information scores were predicted by being scheduled to be operated on within 24 h and operated in a governmental hospital. Details of the multiple linear regression are shown in Table 3.

**Table 3:** Details of the multiple linear regression with APAIS scores

Domain	Variable	Unstandardized coefficients	SE	Standardized coefficients	*t*	*p* value
APAIS total score	Gender	2.94	0.72	0.25	4.10	< 0.001
	Age	− 0.95	0.73	− 0.08	− 1.29	0.196
	Marital status	1.09	0.77	0.08	1.42	0.158
	Employment status	0.51	0.69	0.04	0.74	0.458
	Presence of chronic disease	0.95	0.75	0.08	1.27	0.204
	Timing of the scheduled surgery	− 2.35	0.74	− 0.18	− 3.17	0.002
	Type of anesthesia to be used in the scheduled surgery	− 1.71	0.93	− 0.10	− 1.84	0.067
	Have had surgical complications	2.72	1.08	0.14	2.51	0.013
APAIS anxiety score	Gender	2.37	0.52	0.27	4.60	< 0.001
	Age	− 0.56	0.53	− 0.06	− 1.05	0.293
	Marital status	0.83	0.55	0.08	1.50	0.136
	Employment status	0.75	0.50	0.09	1.52	0.131
	Presence of chronic disease	1.12	0.54	0.13	2.08	0.038
	Timing of the scheduled surgery	− 1.46	0.53	− 0.15	− 2.73	0.007
	Type of anesthesia to be used in the scheduled surgery	− 0.83	0.67	− 0.07	− 1.24	0.215
	Have had surgical complications	2.03	0.78	0.14	2.60	0.010
APAIS need for information score	Gender	0.56	0.32	0.10	1.75	0.082
	Timing of the scheduled surgery	− 0.91	0.36	− 0.15	− 2.53	0.012
	Type of anesthesia to be used in the scheduled surgery	− 0.84	0.45	− 0.11	− 1.88	0.062
	Hospital where the surgery will be performed	− 0.66	0.33	− 0.12	− 2.01	0.045

To control potentially confounding factors, the variables that were significantly associated in the Chi-square or Fisher’s exact tests were included in multivariate logistic regression models. Female patients were 2.89 times (95% CI 1.44–5.81) more likely to express high anxiety compared to male patients. On the other hand, patients to be operated within 24 h, scheduled to receive general or regional anesthesia, and those to be operated in governmental hospitals were 2.04-times (95% CI 1.15–3.60), 2.36-times (95% CI 1.14–4.88), and 2.08-times (1.24–3.50) compared to those who would be operated after 24 h, scheduled to receive local anesthesia, and those to be operated in private hospitals. Details of the multivariate logistic regression models are shown in Table 4.

**Table 4:** Details of the multivariate logistic regression models with APAIS categories

							95% CI for OR	
Domain	Variable	β	SE	Wald	*p* value	OR	Lower	Upper
APAIS anxiety	Gender	1.06	0.36	8.92	0.003	2.89	1.44	5.81
	Age	0.27	0.34	0.65	0.419	1.31	0.68	2.53
	Marital status	0.81	0.41	3.92	0.048	2.24	1.01	4.97
	Employment status	− 0.29	0.32	0.80	0.370	0.75	0.40	1.41
	Presence of chronic disease	− 0.68	0.36	3.58	0.058	0.51	0.25	1.02
	Timing of the scheduled surgery	0.76	0.39	3.71	0.054	2.14	0.99	4.64
	Type of anesthesia to be used in the scheduled surgery	0.68	0.53	1.63	0.202	1.97	0.69	5.61
	Have had surgical complications	− 0.90	0.47	3.74	0.053	0.41	0.16	1.01
APAIS need for information	Gender	0.49	0.26	3.60	0.058	1.64	0.98	2.72
	Timing of the scheduled surgery	0.71	0.29	5.98	0.014	2.04	1.15	3.60
	Type of anesthesia to be used in the scheduled surgery	0.86	0.37	5.32	0.021	2.36	1.14	4.88
	Hospital where the surgery will be performed	0.73	0.27	7.63	0.006	2.08	1.24	3.50

### STAIS-5/STAIT-5 scores

The mean STAIS-5 score was 10.0 ± 4.2 and the mean STAIT-5 was 10.3 ± 3.8. Of the patients, 140 (50.0%) had high state anxiety and 56 (20.0%) had high trait anxiety. The detailed responses of the patients are shown in Table 5.

**Table 5:** Detailed responses of the patients on the STAIS-5/STAIT-5 items

			Not at all		Somewhat		Moderately		Very much	
	#	Item	*n*	%	*n*	%	*n*	%	*n*	%
STAIS-5	1	I feel upset	91	32.5	92	32.9	63	22.5	34	12.1
	2	I feel frightened	140	50.0	66	23.6	41	14.6	33	11.8
	3	I feel nervous	94	33.6	91	32.5	55	19.6	40	14.3
	4	I am jittery	132	47.1	73	26.1	41	14.6	34	12.1
	5	I feel confused	130	46.4	71	25.4	41	14.6	38	13.6
STAIT-5	1	I feel that difficulties are piling up so that I cannot overcome them	107	38.2	88	31.4	54	19.3	31	11.1
	2	I worry too much over something that really doesn't matter	122	43.6	69	24.6	49	17.5	40	14.3
	3	Some unimportant thoughts run through my mind and bothers me	93	33.2	83	29.6	52	18.6	52	18.6
	4	I take disappointments so keenly that I can't put them out of my mind	156	55.7	53	18.9	30	10.7	41	14.6
	5	I get in a state of tension or turmoil as I think over my recent concerns and interests	101	36.1	79	28.2	58	20.7	42	15.0

### Association between STAIS-5 and STAIT-5 scores with demographic, clinical, and surgical variables of the patients 

The *t* tests, ANOVA, chi-square, and Fisher’s exact tests showed that STAIS-5 scores were significantly higher for patients who were female, younger than 42 years, married, employed, scheduled to be operated on within 24 h, and scheduled to receive either obstetrical/gynecological, ophthalmological, general, or ear, nose, and throat operations. On the other hand, the STAIT-5 scores were significantly higher for patients who were female, scheduled to receive general or regional anesthesia, and scheduled to receive either obstetrical/gynecological, cardiac surgery/interventions, ophthalmological, general, or ear, nose, and throat operations. Differences in STAIS-5 and STAIT-5 scores are shown in Supplementary Table S3 and associations between demographic, clinical, and surgical variables of the patients with anxiety and need for information categories are shown in Supplementary Table S4.

To control potentially confounding factors, the variables that were significantly associated in the t-tests and ANOVA were included in multiple linear regression models. The models showed that higher STAIS-5 scores were predicted by being female, younger than 42 years, and scheduled to be operated on within 24 h. On the other hand, higher STAIT-5 scores were predicted by being female. Details of the multiple linear regression are shown in Table 6.

**Table 6:** Details of the multiple linear regression with STAIS-5 and STAIT-5 scores

Domain	Variable	Unstandardized coefficients	SE	Standardized coefficients	*t*	*p* value
STAIS-5 score	Gender	2.13	0.52	0.26	4.13	< 0.001
	Age	− 1.21	0.47	− 0.15	− 2.59	0.010
	Marital status	1.16	0.55	0.12	2.10	0.036
	Employment status	0.34	0.50	0.04	0.68	0.498
	Timing of the scheduled surgery	− 1.29	0.54	− 0.14	− 2.41	0.017
STAIT-5 score	Gender	2.01	0.44	0.26	4.57	< 0.001
	Type of anesthesia to be used in the scheduled surgery	− 1.01	0.62	− 0.09	− 1.63	0.105

To control potentially confounding factors, the variables that were significantly associated in the Chi-square or Fisher’s exact tests were included in multivariate logistic regression models. Patients who were female, younger than 42 years, and married were 2.61-times (95% CI 1.53–4.46), 1.90-times (95% CI 1.14–3.15), and 2.02-times (95% CI 1.11–3.66) more likely to express high state anxiety compared male, 42 years and older, and single patients, respectively. On the other hand, the patients who were female and those who were younger than 42 years were 1.99-times (95% CI 1.06–3.71) and 1.95-times (95% CI 1.06–3.60) more likely to express high trait anxiety compared to male and 42 years and older patients, respectively. Details of the multivariate logistic regression models are shown in Table 7.

**Table 7:** Details of the multivariate logistic regression models with STAIS-5 and STAIT-5 categories

							95% CI for OR	
Domain	Variable	β	SE	Wald	*p* value	OR	Lower	Upper
STAIS-5	Gender	0.96	0.27	12.29	< 0.001	2.61	1.53	4.46
	Age	0.64	0.26	6.13	0.013	1.90	1.14	3.15
	Marital status	0.70	0.30	5.29	0.021	2.02	1.11	3.66
	Employment status	− 0.25	0.27	0.88	0.349	0.78	0.46	1.32
STAIT-5	Gender	0.69	0.32	4.64	0.031	1.99	1.06	3.71
	Age	0.67	0.31	4.61	0.032	1.95	1.06	3.60

### Correlation between APAIS total, APAIS anxiety, APAIS need for information, STAIS-5, and STAIT-5 scores

There was a strong and positive correlation between APAIS total scores with APAIS anxiety scores (Pearson’s *r* = 0.90, *p* value < 0.001), and APAIS need for information scores (Pearson’s *r* = 0.74, *p* value < 0.001). On the other hand, there was a moderate and positive correlation between APAIS total scores and STAIS-5 scores (Pearson’s *r* = 0.60, *p* value < 0.001) and STAIT-5 scores (Pearson’s *r* = 0.41, *p* value < 0.001). There was a moderate correlation between APAIS anxiety scores and APAIS need for information scores (Pearson’s *r* = 0.39, *p* value < 0.001), STAIS-5 scores (Pearson’s *r* = 0.65, *p* value < 0.001) and STAIT-5 scores (Pearson’s *r* = 0.40, *p* value < 0.001). Similarly, there was a weak and positive correlation between APAIS need for information scores and STAIS-5 scores (Pearson’s *r* = 0.28, *p* value < 0.001) and STAIT-5 scores (Pearson’s *r* = 0.27, *p* value < 0.001). Moreover, there was a moderate and positive correlation between STAIS-5 scores and STAIT-5 scores (Pearson’s *r* = 0.46, *p* value < 0.001).

## Discussion 

Improving the care of patients in perioperative care has been prioritized in different healthcare systems around the world (Tola et al. 2021). For the first time, this study assessed the prevalence of perioperative anxiety among patients scheduled for surgical operations/interventions under anesthesia in Palestine. Additionally, the study identified the factors associated with higher anxiety. The findings of this study could be informative to decision and policymakers in healthcare authorities, anesthesiologists, and other providers of perioperative care who might need to design and implement measures to reduce preoperative anxiety and improve perioperative care in developing countries.

Using the APAIS scale, the prevalence of high preoperative anxiety was 27.1%. On the other hand, the prevalence of a high need for information was 57.1%. Moreover, the prevalence of high state anxiety was 50% and the prevalence of high trait anxiety was 20%. Recent systematic reviews reported that the prevalence rates of preoperative anxiety ranged from 17 to 89% (Bedaso et al. 2022; Oteri et al. 2021; Friedrich et al. 2022). The prevalence rate reported in this study was comparable to that reported in a recent large-scale multicenter study in China despite using a different scale to measure preoperative anxiety (Li et al. 2021). These findings were not surprising as the different scales measured the same constructs. In this study, scores obtained in the APAIS, STAIS-5, and STAIT-5 correlated positively. On the other hand, the prevalence of preoperative anxiety reported in this study was less than that reported in a recent study in Germany (Aust et al. 2018). Similarly, the prevalence rate reported in this study was lower than the reported global pooled rate of 48% (Friedrich et al. 2022). Another systematic review with meta-analysis reported a prevalence rate of preoperative anxiety of 55.7% in low- and middle-income countries (Bedaso et al. 2022). Taken together, these findings indicate that the prevalence rate of preoperative anxiety can vary significantly by the tools used to measure preoperative anxiety as well as patient- and healthcare-system-related factors (Bedaso et al. 2022; Aust et al. 2018; Gürler et al. 2022; Yu et al. 2022; Friedrich et al. 2022). Therefore, decision and policymakers need to consider the tools used to assess preoperative anxiety as well as the patient- and healthcare-system-related factors when interpreting and considering prevalence rates of preoperative anxiety.

In this study, preoperative anxiety scores were higher for female patients. These findings were consistent with those reported in previous studies that were conducted in different healthcare systems (Bedaso et al. 2022; Navarro-Gastón and Munuera-Martínez 2020; Yu et al. 2022; Oteri et al. 2021; Friedrich et al. 2022). Probably, anesthesiologists and other providers of perioperative care need to consider the sex of the patient when planning preoperative counseling. Additionally, the findings of this study showed that patients who had chronic diseases reported higher anxiety compared to those who did not have chronic diseases. These findings were not surprising because it is well-established that chronic diseases can deteriorate the outcomes of surgical interventions. A recent study that analyzed data from the English National Health System showed that the presence of chronic disease was associated with a 10 times higher risk of postoperative mortality among surgical patients (Fowler et al. 2022). Probably, patients with chronic diseases feared death or the occurrence of postoperative complications as a result of their pre-existing health conditions. Therefore, decision-makers, anesthesiologists, and other providers of perioperative care should consider providing tailored counseling to patients with chronic diseases. Moreover, having experienced surgical complications was a predictor of higher anxiety among preoperative patients. These findings were consistent with those reported in previous studies (Li et al. 2021; Friedrich et al. 2022). These studies showed that higher anxiety was associated with the degree of surgical invasiveness. Taken together, providers of perioperative care should provide more information to patients who have had surgical complications and the measures that would be taken to avoid the occurrence of these complications. Again, this study showed that anxiety increased as the scheduled operation approached. These findings indicate that providers of perioperative care should intensify measures to reduce preoperative anxiety in the hours that precede the surgical intervention. Previous studies have shown that some audiovisual interventions were effective in reducing preoperative anxiety (Chow et al. 2016). Anesthesiologists and other providers of perioperative care might use effective interventions to reduce preoperative anxiety.

### Strengths and limitations

The findings of this study might be interpreted after considering the following strengths and limitations. First, this is the first study to assess the prevalence of preoperative anxiety among patients in the Palestinian healthcare system. Additionally, the study identified the predictors of high anxiety. The findings of this study might be used by decision and policymakers, anesthesiologists, and other providers of perioperative care to design and implement measures to reduce preoperative anxiety, especially among those with risk factors. Second, the patients included in this study were diversified in terms of demographic, clinical, and surgical variables. The study patients were recruited from different hospitals. This diversity should have improved the representativeness of the entire population of preoperative patients and should have improved the external validity of the study. Third, anxiety was measured using different validated and reliable scales. These scales assessed the different domains of anxiety. This should have added depth and width to the findings reported in this study. Fourth, the questionnaire was tested in a pilot study before it was used in the larger study. Additionally, the test–retest reliability and internal consistency were used as diagnostics for the suitability of the questionnaire. These steps should have ensured the generation of valid and reliable findings.

On the other hand, this study had some limitations. First, this study was conducted using an observational design. The use of an intervention to reduce preoperative anxiety should have produced more interesting findings. Second, the sample size was calculated using a 90% CI. A larger sample size could have produced more reliable findings. Third, the results of this study were self-reported as in this study no somatic signs and symptoms were used to assess anxious patients. Fourth, desirability bias could not be excluded as some patients might have tended to provide more positive answers or hide their anxiety. In this case, the prevalence of preoperative anxiety might have been underestimated. Finally, this study was conducted in Palestine. Studies conducted in more than one country should report more interesting findings.

## Conclusion

Preoperative anxiety was prevalent among patients scheduled for surgical operations in Palestinian hospitals. Anesthesiologists and other providers of perioperative care should screen preoperative patients who are female, have chronic diseases, are scheduled to be operated on within 24 h, and having had experienced surgical complications for preoperative anxiety. The findings reported in this study could be used by decision and policymakers in developing countries to plan, design, and implement effective interventions to reduce preoperative anxiety among surgical patients. More studies are still needed to investigate the effects of the implemented measures on the prevalence of preoperative anxiety.

### Supplementary Information

**Additional file 1: Supplementary Table S1.** Differences in APAIS scores.

**Additional file 2: Supplementary Table S2.** Associations between demographic, clinical, and surgical variables of the patients with APAIS anxiety and need for information categories.

**Additional file 3: Supplementary Table S3.** Differences in STAIS-5 and STAIT-5 scores.

**Additional file 4: Supplementary Table S4.** Associations between demographic, clinical, and surgical variables of the patients with STAIS-5 and STAIT-5 categories.

## Data Availability

All data relevant to this study were included in the results or provided as supplementary materials with this manuscript.
