# Supplementary material for: Prevalence of preoperative anxiety among hospitalized patients in a developing country: a study of associated factors
Source: Perioper Med (Lond). 2023 Aug 24;12:47. doi: 10.1186/s13741-023-00336-w (PMC10463373; doi:10.1186/s13741-023-00336-w)
Supplement: Supplementary file 1 — Additional file 1: Supplementary Table S1. Differences in APAIS scores. [file 13741_2023_336_MOESM1_ESM.docx]

**Supplementary Table S1:** Differences in APAIS scores

|  |  |  | **APAIS total score** | | | **APAIS anxiety score** | | | **APAIS need for information score** | | |
| --- | --- | --- | --- | --- | --- | --- | --- | --- | --- | --- | --- |
| **Variable** | **n** | **%** | **Mean** | **SD** | **p-value** | **Mean** | **SD** | **p-value** | **Mean** | **SD** | **p-value** |
| **Gender** |  |  |  |  |  |  |  |  |  |  |  |
| Male | 128 | 45.7 | 11.4 | 4.4 | < 0.001 | 6.6 | 2.9 | < 0.001 | 4.9 | 2.5 | 0.012 |
| Female | 152 | 54.3 | 15.5 | 6.4 |  | 9.9 | 4.7 |  | 5.7 | 2.8 |  |
| **Age (years)** |  |  |  |  |  |  |  |  |  |  |  |
| < 42 | 142 | 50.7 | 14.5 | 5.8 | 0.012 | 9.0 | 4.3 | 0.011 | 5.5 | 2.6 | 0.147 |
| ≥ 42 | 138 | 49.3 | 12.8 | 5.8 |  | 7.7 | 4.3 |  | 5.1 | 2.7 |  |
| **Marital status** |  |  |  |  |  |  |  |  |  |  |  |
| Single (never married) | 73 | 26.1 | 12.1 | 4.6 | 0.011 | 7.1 | 3.0 | 0.005 | 5.0 | 2.6 | 0.287 |
| Was married (currently married/divorced/widowed) | 207 | 73.9 | 14.2 | 6.2 |  | 8.8 | 4.6 |  | 5.4 | 2.7 |  |
| **Educational level** |  |  |  |  |  |  |  |  |  |  |  |
| School | 197 | 70.4 | 13.3 | 5.8 | 0.108 | 8.0 | 4.3 | 0.067 | 5.2 | 2.8 | 0.554 |
| University | 83 | 29.6 | 14.5 | 6.0 |  | 9.1 | 4.2 |  | 5.4 | 2.5 |  |
| **Employment status** |  |  |  |  |  |  |  |  |  |  |  |
| Unemployed | 142 | 50.7 | 12.8 | 5.2 | 0.011 | 7.5 | 3.6 | 0.001 | 5.3 | 2.7 | 0.908 |
| Employed | 138 | 49.3 | 14.6 | 6.4 |  | 9.2 | 4.8 |  | 5.3 | 2.7 |  |
| **Place of residence** |  |  |  |  |  |  |  |  |  |  |  |
| Rural | 114 | 40.7 | 13.1 | 6.1 | 0.234 | 8.1 | 4.3 | 0.345 | 5.1 | 2.6 | 0.271 |
| Urban | 166 | 59.3 | 14.0 | 5.8 |  | 8.5 | 4.3 |  | 5.4 | 2.7 |  |
| **Self-rated satisfaction with household income** | |  |  |  |  |  |  |  |  |  |  |
| Low | 34 | 12.1 | 12.4 | 0.8 | 0.342 | 7.5 | 0.6 | 0.404 | 4.9 | 0.4 | 0.558 |
| Moderate | 234 | 83.6 | 13.8 | 0.4 |  | 8.4 | 0.3 |  | 5.4 | 0.2 |  |
| High | 12 | 4.3 | 14.8 | 1.7 |  | 9.3 | 1.2 |  | 5.6 | 0.7 |  |
| **Self-rated satisfaction with social life** |  |  |  |  |  |  |  |  |  |  |  |
| Low | 15 | 5.4 | 14.7 | 1.7 | 0.733 | 8.5 | 1.2 | 0.966 | 6.1 | 0.7 | 0.232 |
| Moderate | 155 | 55.4 | 13.5 | 0.5 |  | 8.4 | 0.4 |  | 5.1 | 0.2 |  |
| High | 110 | 39.3 | 13.8 | 0.5 |  | 8.3 | 0.4 |  | 5.5 | 0.3 |  |
| **Self-rated satisfaction with religious commitment** | | |  |  |  |  |  |  |  |  |  |
| Low | 10 | 3.6 | 14.4 | 1.2 | 0.283 | 8.7 | 1.2 | 0.764 | 5.7 | 0.8 | 0.065 |
| Moderate | 156 | 55.7 | 14.1 | 0.5 |  | 8.5 | 0.3 |  | 5.6 | 0.2 |  |
| High | 114 | 40.7 | 13.0 | 0.6 |  | 8.1 | 0.4 |  | 4.9 | 0.3 |  |
| **Presence of chronic disease** |  |  |  |  |  |  |  |  |  |  |  |
| No | 105 | 37.5 | 12.5 | 5.1 | 0.012 | 7.3 | 3.6 | 0.002 | 5.2 | 2.7 | 0.564 |
| Yes | 175 | 62.5 | 14.3 | 6.2 |  | 9.0 | 4.6 |  | 5.4 | 2.7 |  |
| **Timing of the scheduled surgery** |  |  |  |  |  |  |  |  |  |  |  |
| Within ≤ 24 h | 206 | 73.6 | 14.5 | 6.0 | < 0.001 | 8.9 | 4.5 | < 0.001 | 5.6 | 2.6 | 0.004 |
| > 24 h | 74 | 26.4 | 11.3 | 4.7 |  | 6.8 | 3.3 |  | 4.5 | 2.7 |  |
| **Type of anesthesia to be used in the scheduled surgery** | | |  |  |  |  |  |  |  |  |  |
| General/regional anesthesia | 239 | 85.4 | 14.1 | 6.0 | 0.003 | 8.6 | 4.4 | 0.014 | 5.5 | 2.7 | 0.010 |
| Local anesthesia | 41 | 14.6 | 11.1 | 4.9 |  | 6.8 | 3.1 |  | 4.3 | 2.5 |  |
| **Hospital where the surgery will be performed** | |  |  |  |  |  |  |  |  |  |  |
| Governmental | 173 | 61.8 | 14.0 | 5.6 | 0.202 | 8.4 | 4.2 | 0.796 | 5.6 | 2.6 | 0.016 |
| Private | 107 | 38.2 | 13.1 | 6.3 |  | 8.3 | 4.5 |  | 4.8 | 2.7 |  |
| **Have had previous surgery** |  |  |  |  |  |  |  |  |  |  |  |
| No | 86 | 30.7 | 14.6 | 6.1 | 0.085 | 9.0 | 4.5 | 0.091 | 5.6 | 2.5 | 0.282 |
| Yes | 194 | 69.3 | 13.2 | 5.8 |  | 8.1 | 4.2 |  | 5.2 | 2.7 |  |
| **Have had surgical complications** |  |  |  |  |  |  |  |  |  |  |  |
| No | 253 | 90.4 | 13.3 | 5.7 | 0.009 | 8.1 | 4.2 | 0.010 | 5.2 | 2.6 | 0.113 |
| Yes | 27 | 9.6 | 16.4 | 6.6 |  | 10.4 | 4.9 |  | 6.1 | 3.1 |  |
| **Type of surgery** |  |  |  |  |  |  |  |  |  |  |  |
| General | 84 | 30.0 | 14.3 | 0.7 | < 0.001 | 8.4 | 0.5 | < 0.001 | 5.9 | 0.3 | 0.003 |
| Obstetrics and gynecology | 63 | 22.5 | 16.5 | 0.8 |  | 10.9 | 0.6 |  | 5.5 | 0.4 |  |
| Orthopedic | 44 | 15.7 | 12.4 | 0.7 |  | 7.2 | 0.6 |  | 5.2 | 0.4 |  |
| Ear, nose, and throat | 22 | 7.9 | 14.0 | 1.1 |  | 8.5 | 0.8 |  | 5.4 | 0.5 |  |
| Urology | 25 | 8.9 | 10.6 | 1.0 |  | 6.3 | 0.7 |  | 4.4 | 0.5 |  |
| Ophthalmology | 4 | 1.4 | 15.5 | 5.0 |  | 8.5 | 3.8 |  | 7.0 | 1.7 |  |
| Neurosurgery | 15 | 5.4 | 11.9 | 0.9 |  | 6.7 | 0.6 |  | 5.2 | 0.5 |  |
| Cardiac surgery/intervention | 15 | 5.4 | 9.6 | 0.7 |  | 6.5 | 0.5 |  | 3.1 | 0.4 |  |
| Minor surgeries/interventions | 8 | 2.9 | 9.9 | 1.6 |  | 6.1 | 1.0 |  | 3.8 | 0.8 |  |
