# Supplementary material for: Prevalence of preoperative anxiety among hospitalized patients in a developing country: a study of associated factors
Source: Perioper Med (Lond). 2023 Aug 24;12:47. doi: 10.1186/s13741-023-00336-w (PMC10463373; doi:10.1186/s13741-023-00336-w)
Supplement: Supplementary file 2 — Additional file 2: Supplementary Table S2. Associations between demographic, clinical, and surgical variables of the patients with APAIS anxiety and need for information categories. [file 13741_2023_336_MOESM2_ESM.docx]

**Supplementary Table S2:** Associations between demographic, clinical, and surgical variables of the patients with APAIS anxiety and need for information categories

|  |  |  | **APAIS anxiety category** | | | | | | **APAIS need for information category** | | | | | |
| --- | --- | --- | --- | --- | --- | --- | --- | --- | --- | --- | --- | --- | --- | --- |
|  |  |  | **No-Minimal anxiety** | | **High anxiety** | |  |  | **Little need for information** | | **High need for information** | |  |  |
| **Variable** | **N** | **%** | **n** | **%** | **n** | **%** | **Chi-square/FET** | **p-value** | **n** | **%** | **n** | **%** | **Chi-square/FET** | **p-value** |
| **Gender** |  |  |  |  |  |  |  |  |  |  |  |  |  |  |
| Male | 128 | 45.7 | 112 | 40.0 | 16 | 5.7 | 25.6 | < 0.001 | 66 | 23.6 | 62 | 22.1 | 7.3 | 0.008 |
| Female | 152 | 54.3 | 92 | 32.9 | 60 | 21.4 |  |  | 54 | 19.3 | 98 | 35.0 |  |  |
| **Age (years)** |  |  |  |  |  |  |  |  |  |  |  |  |  |  |
| < 42 | 142 | 50.7 | 95 | 33.9 | 47 | 16.8 | 5.2 | 0.031 | 56 | 20.0 | 86 | 30.7 | 1.4 | 0.277 |
| ≥ 42 | 138 | 49.3 | 109 | 38.9 | 29 | 10.4 |  |  | 64 | 22.9 | 74 | 26.4 |  |  |
| **Marital status** |  |  |  |  |  |  |  |  |  |  |  |  |  |  |
| Single (never married) | 73 | 26.1 | 63 | 22.5 | 141 | 50.4 | 9.0 | 0.003 | 35 | 12.5 | 85 | 30.4 | 1.0 | 0.337 |
| Was married (currently married/divorced/widowed) | 207 | 73.9 | 10 | 3.6 | 66 | 23.6 |  |  | 38 | 13.6 | 122 | 43.6 |  |  |
| **Educational level** |  |  |  |  |  |  |  |  |  |  |  |  |  |  |
| School | 197 | 70.4 | 150 | 53.6 | 54 | 19.3 | 3.6 | 0.077 | 86 | 30.7 | 34 | 12.1 | 0.2 | 0.694 |
| University | 83 | 29.6 | 47 | 16.8 | 29 | 10.4 |  |  | 111 | 39.6 | 49 | 17.5 |  |  |
| **Employment status** |  |  |  |  |  |  |  |  |  |  |  |  |  |  |
| Unemployed | 142 | 50.7 | 113 | 40.4 | 91 | 32.5 | 6.6 | 0.011 | 63 | 22.5 | 57 | 20.4 | 0.3 | 0.630 |
| Employed | 138 | 49.3 | 29 | 10.4 | 47 | 16.8 |  |  | 79 | 28.2 | 81 | 28.9 |  |  |
| **Place of residence** |  |  |  |  |  |  |  |  |  |  |  |  |  |  |
| Rural | 114 | 40.7 | 85 | 30.4 | 119 | 42.5 | 0.3 | 0.682 | 52 | 18.6 | 68 | 24.3 | 0.6 | 0.463 |
| Urban | 166 | 59.3 | 29 | 10.4 | 47 | 16.8 |  |  | 62 | 22.1 | 98 | 35.0 |  |  |
| **Self-rated satisfaction with household income** | | |  |  |  |  |  |  |  |  |  |  |  |  |
| Low | 34 | 12.1 | 27 | 9.6 | 7 | 2.5 | 0.9 | 0.638 | 15 | 5.4 | 19 | 6.8 | 0.0 | 1.000 |
| Moderate | 234 | 83.6 | 168 | 60.0 | 66 | 23.6 |  |  | 100 | 35.7 | 134 | 47.9 |  |  |
| High | 12 | 4.3 | 9 | 3.2 | 3 | 1.1 |  |  | 5 | 1.8 | 7 | 2.5 |  |  |
| **Self-rated satisfaction with social life** | | |  |  |  |  |  |  |  |  |  |  |  |  |
| Low | 15 | 5.4 | 10 | 3.6 | 5 | 1.8 | 1.3 | 0.590 | 5 | 1.8 | 10 | 3.6 | 0.6 | 0.793 |
| Moderate | 155 | 55.4 | 110 | 39.3 | 45 | 16.1 |  |  | 67 | 23.9 | 88 | 31.4 |  |  |
| High | 110 | 39.3 | 84 | 30.0 | 26 | 9.3 |  |  | 48 | 17.1 | 62 | 22.1 |  |  |
| **Self-rated satisfaction with religious commitment** | | |  |  |  |  |  |  |  |  |  |  |  |  |
| Low | 10 | 3.6 | 7 | 2.5 | 3 | 1.1 | 0.3 | 0.885 | 4 | 1.4 | 6 | 2.1 | 4.0 | 0.134 |
| Moderate | 156 | 55.7 | 112 | 40.0 | 44 | 15.7 |  |  | 59 | 21.1 | 97 | 34.6 |  |  |
| High | 114 | 40.7 | 85 | 30.4 | 29 | 10.4 |  |  | 57 | 20.4 | 57 | 20.4 |  |  |
| **Presence of chronic disease** | |  |  |  |  |  |  |  |  |  |  |  |  |  |
| No | 105 | 37.5 | 87 | 31.1 | 18 | 6.4 | 8.5 | 0.004 | 47 | 16.8 | 58 | 20.7 | 0.2 | 0.618 |
| Yes | 175 | 62.5 | 117 | 41.8 | 58 | 20.7 |  |  | 73 | 26.1 | 102 | 36.4 |  |  |
| **Timing of the scheduled surgery** | |  |  |  |  |  |  |  |  |  |  |  |  |  |
| Within ≤ 24 h | 206 | 73.6 | 141 | 50.4 | 65 | 23.2 | 7.7 | 0.006 | 78 | 27.9 | 128 | 45.7 | 7.9 | 0.006 |
| > 24 h | 74 | 26.4 | 63 | 22.5 | 11 | 3.9 |  |  | 42 | 15.0 | 32 | 11.4 |  |  |
| **Type of anesthesia to be used in the scheduled surgery** | | |  |  |  |  |  |  |  |  |  |  |  |  |
| General/regional anesthesia | 239 | 85.4 | 168 | 60.0 | 71 | 25.4 | 5.4 | 0.022 | 93 | 33.2 | 146 | 52.1 | 10.4 | 0.002 |
| Local anesthesia | 41 | 14.6 | 36 | 12.9 | 5 | 1.8 |  |  | 27 | 9.6 | 14 | 5.0 |  |  |
| **Hospital where the surgery will be performed** | | |  |  |  |  |  |  |  |  |  |  |  |  |
| Governmental | 173 | 61.8 | 123 | 43.9 | 50 | 17.9 | 0.7 | 0.411 | 61 | 21.8 | 112 | 40.0 | 10.7 | 0.001 |
| Private | 107 | 38.2 | 81 | 28.9 | 26 | 9.3 |  |  | 59 | 21.1 | 48 | 17.1 |  |  |
| **Have had previous surgery** |  |  |  |  |  |  |  |  |  |  |  |  |  |  |
| No | 86 | 30.7 | 58 | 20.7 | 28 | 10.0 | 1.8 | 0.191 | 35 | 12.5 | 51 | 18.2 | 0.2 | 0.695 |
| Yes | 194 | 69.3 | 146 | 52.1 | 48 | 17.1 |  |  | 85 | 30.4 | 109 | 38.9 |  |  |
| **Have had surgical complications** | |  |  |  |  |  |  |  |  |  |  |  |  |  |
| No | 253 | 90.4 | 189 | 67.5 | 64 | 22.9 | 4.5 | 0.041 | 110 | 39.3 | 143 | 51.1 | 0.4 | 0.548 |
| Yes | 27 | 9.6 | 15 | 5.4 | 12 | 4.3 |  |  | 10 | 3.6 | 17 | 6.1 |  |  |
| **Type of surgery** |  |  |  |  |  |  |  |  |  |  |  |  |  |  |
| General | 84 | 30.0 | 56 | 20.0 | 28 | 10.0 | 22.6 | < 0.001 | 27 | 28.0 | 57 | 28.0 | 10.6 | 0.001 |
| Obstetrics and gynecology | 63 | 22.5 | 30 | 10.7 | 33 | 11.8 |  |  | 25 | 28.0 | 38 | 28.0 |  |  |
| Orthopedic | 44 | 15.7 | 36 | 12.9 | 8 | 2.9 |  |  | 18 | 28.0 | 26 | 28.0 |  |  |
| Ear, nose, and throat | 22 | 7.9 | 19 | 6.8 | 3 | 1.1 |  |  | 11 | 28.0 | 11 | 28.0 |  |  |
| Urology | 25 | 8.9 | 24 | 8.6 | 1 | 0.4 |  |  | 17 | 28.0 | 8 | 28.0 |  |  |
| Ophthalmology | 4 | 1.4 | 3 | 1.1 | 1 | 0.4 |  |  | 1 | 28.0 | 3 | 28.0 |  |  |
| Neurosurgery | 15 | 5.4 | 14 | 5.0 | 1 | 0.4 |  |  | 3 | 28.0 | 12 | 28.0 |  |  |
| Cardiac surgery/intervention | 15 | 5.4 | 15 | 5.4 | 0 | 0.0 |  |  | 12 | 28.0 | 3 | 28.0 |  |  |
| Minor surgeries/interventions | 8 | 2.9 | 7 | 2.5 | 1 | 0.4 |  |  | 6 | 28.0 | 2 | 28.0 |  |  |
