# Supplementary material for: Prevalence of preoperative anxiety among hospitalized patients in a developing country: a study of associated factors
Source: Perioper Med (Lond). 2023 Aug 24;12:47. doi: 10.1186/s13741-023-00336-w (PMC10463373; doi:10.1186/s13741-023-00336-w)
Supplement: Supplementary file 3 — Additional file 3: Supplementary Table S3. Differences in STAIS-5 and STAIT-5 scores. [file 13741_2023_336_MOESM3_ESM.docx]

**Supplementary Table S3:** Differences in STAIS-5 and STAIT-5 scores

|  |  |  | **STAIS-5 score** | | | **STAIT-5 score** | | |
| --- | --- | --- | --- | --- | --- | --- | --- | --- |
| **Variable** | **n** | **%** | **Mean** | **SD** | **p-value** | **Mean** | **SD** | **p-value** |
| **Gender** |  |  |  |  |  |  |  |  |
| Male | 128 | 45.7 | 8.5 | 3.5 | < 0.001 | 9.1 | 3.6 | < 0.001 |
| Female | 152 | 54.3 | 11.3 | 4.3 |  | 11.2 | 3.8 |  |
| **Age (years)** |  |  |  |  |  |  |  |  |
| < 42 | 142 | 50.7 | 10.7 | 4.3 | 0.009 | 10.4 | 3.9 | 0.662 |
| ≥ 42 | 138 | 49.3 | 9.4 | 3.9 |  | 10.2 | 3.7 |  |
| **Marital status** |  |  |  |  |  |  |  |  |
| Single (never married) | 73 | 26.1 | 8.8 | 3.5 | 0.005 | 9.7 | 3.9 | 0.147 |
| Was married (currently married/divorced/widowed) | 207 | 73.9 | 10.4 | 4.3 |  | 10.5 | 3.8 |  |
| **Educational level** |  |  |  |  |  |  |  |  |
| School | 197 | 70.4 | 9.8 | 4.1 | 0.146 | 10.5 | 3.8 | 0.177 |
| University | 83 | 29.6 | 10.6 | 4.3 |  | 9.8 | 3.7 |  |
| **Employment status** |  |  |  |  |  |  |  |  |
| Unemployed | 142 | 50.7 | 9.4 | 4.0 | 0.005 | 9.9 | 3.8 | 0.073 |
| Employed | 138 | 49.3 | 10.7 | 4.3 |  | 10.7 | 3.8 |  |
| **Place of residence** |  |  |  |  |  |  |  |  |
| Rural | 114 | 40.7 | 9.6 | 4.0 | 0.193 | 9.9 | 3.8 | 0.231 |
| Urban | 166 | 59.3 | 10.3 | 4.3 |  | 10.5 | 3.8 |  |
| **Self-rated satisfaction with household income** | | |  |  |  |  |  |  |
| Low | 34 | 12.1 | 9.1 | 0.7 | 0.072 | 9.1 | 0.7 | 0.566 |
| Moderate | 234 | 83.6 | 10.1 | 0.3 |  | 10.1 | 0.3 |  |
| High | 12 | 4.3 | 12.3 | 1.1 |  | 12.3 | 1.1 |  |
| **Self-rated satisfaction with social life** | | |  |  |  |  |  |  |
| Low | 15 | 5.4 | 10.2 | 1.3 | 0.723 | 11.6 | 1.2 | 0.217 |
| Moderate | 155 | 55.4 | 10.2 | 0.3 |  | 10.4 | 0.3 |  |
| High | 110 | 39.3 | 9.8 | 0.4 |  | 9.9 | 0.3 |  |
| **Self-rated satisfaction with religious commitment** | | |  |  |  |  |  |  |
| Low | 10 | 3.6 | 10.7 | 1.4 | 0.708 | 10.7 | 1.3 | 0.652 |
| Moderate | 156 | 55.7 | 9.9 | 0.3 |  | 10.1 | 0.3 |  |
| High | 114 | 40.7 | 10.2 | 0.4 |  | 10.5 | 0.4 |  |
| **Presence of chronic disease** |  |  |  |  |  |  |  |  |
| No | 105 | 37.5 | 9.5 | 4.0 | 0.101 | 10.4 | 3.9 | 0.585 |
| Yes | 175 | 62.5 | 10.3 | 4.2 |  | 10.2 | 3.8 |  |
| **Timing of the scheduled surgery** | |  |  |  |  |  |  |  |
| Within ≤ 24 h | 206 | 73.6 | 10.5 | 4.3 | 0.001 | 10.5 | 3.8 | 0.065 |
| > 24 h | 74 | 26.4 | 8.7 | 3.3 |  | 9.6 | 3.9 |  |
| **Type of anesthesia to be used in the scheduled surgery** | | |  |  |  |  |  |  |
| General/regional anesthesia | 239 | 85.4 | 10.2 | 4.3 | 0.094 | 10.5 | 3.8 | 0.041 |
| Local anesthesia | 41 | 14.6 | 9.0 | 3.4 |  | 9.1 | 3.7 |  |
| **Hospital where the surgery will be performed** | | |  |  |  |  |  |  |
| Governmental | 173 | 61.8 | 9.9 | 4.2 | 0.526 | 10.3 | 3.7 | 0.983 |
| Private | 107 | 38.2 | 10.2 | 4.1 |  | 10.3 | 4.0 |  |
| **Have had previous surgery** |  |  |  |  |  |  |  |  |
| No | 86 | 30.7 | 10.6 | 3.9 | 0.119 | 10.2 | 4.0 | 0.785 |
| Yes | 194 | 69.3 | 9.8 | 4.3 |  | 10.3 | 3.7 |  |
| **Have had surgical complications** | |  |  |  |  |  |  |  |
| No | 253 | 90.4 | 10.0 | 4.2 | 0.329 | 10.2 | 3.8 | 0.140 |
| Yes | 27 | 9.6 | 10.8 | 4.0 |  | 11.3 | 3.5 |  |
| **Type of surgery** |  |  |  |  |  |  |  |  |
| General | 84 | 30.0 | 10.0 | 0.5 | < 0.001 | 10.0 | 0.4 | 0.005 |
| Obstetrics and gynecology | 63 | 22.5 | 12.6 | 0.5 |  | 12.0 | 0.5 |  |
| Orthopedic | 44 | 15.7 | 8.7 | 0.6 |  | 9.6 | 0.6 |  |
| Ear, nose, and throat | 22 | 7.9 | 10.0 | 0.6 |  | 10.3 | 0.8 |  |
| Urology | 25 | 8.9 | 8.4 | 0.6 |  | 8.6 | 0.6 |  |
| Ophthalmology | 4 | 1.4 | 10.0 | 1.6 |  | 8.6 | 0.6 |  |
| Neurosurgery | 15 | 5.4 | 7.6 | 0.5 |  | 9.4 | 0.7 |  |
| Cardiac surgery/intervention | 15 | 5.4 | 8.9 | 0.9 |  | 10.8 | 0.9 |  |
| Minor surgeries/interventions | 8 | 2.9 | 9.6 | 1.6 |  | 9.5 | 1.7 |  |
