# Supplementary material for: Prevalence of preoperative anxiety among hospitalized patients in a developing country: a study of associated factors
Source: Perioper Med (Lond). 2023 Aug 24;12:47. doi: 10.1186/s13741-023-00336-w (PMC10463373; doi:10.1186/s13741-023-00336-w)
Supplement: Supplementary file 4 — Additional file 4: Supplementary Table S4. Associations between demographic, clinical, and surgical variables of the patients with STAIS-5 and STAIT-5 categories. [file 13741_2023_336_MOESM4_ESM.docx]

**Supplementary Table S4:** Associations between demographic, clinical, and surgical variables of the patients with STAIS-5 and STAIT-5 categories

|  |  |  | **STAIS category** | | | | | | **STAIT category** | | | | |  |
| --- | --- | --- | --- | --- | --- | --- | --- | --- | --- | --- | --- | --- | --- | --- |
|  |  |  | **No/minimal state anxiety** | | **High state anxiety** | |  |  | **No/minimal trait anxiety** | | **High trait anxiety** | |  |  |
| **Variable** | **n** | **%** | **n** | **%** | **n** | **%** | **Chi-square/FET** | **p-value** | **n** | **%** | **n** | **%** | **Chi-square/FET** | **p-value** |
| **Gender** |  |  |  |  |  |  |  |  |  |  |  |  |  |  |
| Male | 128 | 45.7 | 84 | 30.0 | 44 | 15.7 | 23.0 | < 0.001 | 110 | 39.3 | 18 | 6.4 | 5.2 | 0.025 |
| Female | 152 | 54.3 | 56 | 20.0 | 96 | 34.3 |  |  | 114 | 40.7 | 38 | 13.6 |  |  |
| **Age (years)** |  |  |  |  |  |  |  |  |  |  |  |  |  |  |
| < 42 | 142 | 50.7 | 61 | 21.8 | 81 | 28.9 | 5.7 | 0.023 | 106 | 37.9 | 36 | 12.9 | 5.2 | 0.025 |
| ≥ 42 | 138 | 49.3 | 79 | 28.2 | 59 | 21.1 |  |  | 118 | 42.1 | 20 | 7.1 |  |  |
| **Marital status** |  |  |  |  |  |  |  |  |  |  |  |  |  |  |
| Single (never married) | 73 | 26.1 | 48 | 17.1 | 92 | 32.9 | 9.8 | 0.003 | 59 | 21.1 | 165 | 58.9 | 0.0 | 0.867 |
| Was married (currently married/divorced/widowed) | 207 | 73.9 | 25 | 8.9 | 115 | 41.1 |  |  | 14 | 5.0 | 42 | 15.0 |  |  |
| **Educational level** |  |  |  |  |  |  |  |  |  |  |  |  |  |  |
| School | 197 | 70.4 | 103 | 36.8 | 37 | 13.2 | 1.4 | 0.295 | 153 | 54.6 | 71 | 25.4 | 2.3 | 0.144 |
| University | 83 | 29.6 | 94 | 33.6 | 46 | 16.4 |  |  | 44 | 15.7 | 12 | 4.3 |  |  |
| **Employment status** |  |  |  |  |  |  |  |  |  |  |  |  |  |  |
| Unemployed | 142 | 50.7 | 83 | 29.6 | 57 | 20.4 | 8.2 | 0.006 | 117 | 41.8 | 107 | 38.2 | 1.0 | 0.370 |
| Employed | 138 | 49.3 | 59 | 21.1 | 81 | 28.9 |  |  | 25 | 8.9 | 31 | 11.1 |  |  |
| **Place of residence** |  |  |  |  |  |  |  |  |  |  |  |  |  |  |
| Rural | 114 | 40.7 | 59 | 21.1 | 81 | 28.9 | 0.2 | 0.715 | 94 | 33.6 | 130 | 46.4 | 0.7 | 0.449 |
| Urban | 166 | 59.3 | 55 | 19.6 | 85 | 30.4 |  |  | 20 | 7.1 | 36 | 12.9 |  |  |
| **Self-rated satisfaction with household income** | | |  |  |  |  |  |  |  |  |  |  |  |  |
| Low | 34 | 12.1 | 21 | 7.5 | 13 | 4.6 | 7.2 | 0.025 | 26 | 9.3 | 8 | 2.9 | 0.5 | 0.769 |
| Moderate | 234 | 83.6 | 117 | 41.8 | 117 | 41.8 |  |  | 189 | 67.5 | 45 | 16.1 |  |  |
| High | 12 | 4.3 | 2 | 0.7 | 10 | 3.6 |  |  | 9 | 3.2 | 3 | 1.1 |  |  |
| **Self-rated satisfaction with social life** | | |  |  |  |  |  |  |  |  |  |  |  |  |
| Low | 15 | 5.4 | 7 | 2.5 | 8 | 2.9 | 0.6 | 0.812 | 9 | 3.2 | 6 | 2.1 | 5.3 | 0.067 |
| Moderate | 155 | 55.4 | 75 | 26.8 | 80 | 28.6 |  |  | 122 | 43.6 | 33 | 11.8 |  |  |
| High | 110 | 39.3 | 58 | 20.7 | 52 | 18.6 |  |  | 93 | 33.2 | 17 | 6.1 |  |  |
| **Self-rated satisfaction with religious commitment** | | |  |  |  |  |  |  |  |  |  |  |  |  |
| Low | 10 | 3.6 | 5 | 1.8 | 5 | 1.8 | 0.1 | 0.975 | 6 | 2.1 | 4 | 1.4 | 4.1 | 0.126 |
| Moderate | 156 | 55.7 | 79 | 28.2 | 77 | 27.5 |  |  | 130 | 46.4 | 26 | 9.3 |  |  |
| High | 114 | 40.7 | 56 | 20.0 | 58 | 20.7 |  |  | 88 | 31.4 | 26 | 9.3 |  |  |
| **Presence of chronic disease** |  |  |  |  |  |  |  |  |  |  |  |  |  |  |
| No | 105 | 37.5 | 57 | 20.4 | 48 | 17.1 | 1.2 | 0.323 | 86 | 30.7 | 19 | 6.8 | 0.4 | 0.644 |
| Yes | 175 | 62.5 | 83 | 29.6 | 92 | 32.9 |  |  | 138 | 49.3 | 37 | 13.2 |  |  |
| **Timing of the scheduled surgery** | |  |  |  |  |  |  |  |  |  |  |  |  |  |
| Within ≤ 24 h | 206 | 73.6 | 97 | 34.6 | 109 | 38.9 | 2.6 | 0.136 | 161 | 57.5 | 45 | 16.1 | 1.7 | 0.237 |
| > 24 h | 74 | 26.4 | 43 | 15.4 | 31 | 11.1 |  |  | 63 | 22.5 | 11 | 3.9 |  |  |
| **Type of anesthesia to be used in the scheduled surgery** | | |  |  |  |  |  |  |  |  |  |  |  |  |
| General/regional anesthesia | 239 | 85.4 | 117 | 41.8 | 122 | 43.6 | 0.7 | 0.499 | 187 | 66.8 | 52 | 18.6 | 3.2 | 0.091 |
| Local anesthesia | 41 | 14.6 | 23 | 8.2 | 18 | 6.4 |  |  | 37 | 13.2 | 4 | 1.4 |  |  |
| **Hospital where the surgery will be performed** | | |  |  |  |  |  |  |  |  |  |  |  |  |
| Governmental | 173 | 61.8 | 90 | 32.1 | 83 | 29.6 | 0.7 | 0.461 | 139 | 49.6 | 34 | 12.1 | 0.0 | 0.879 |
| Private | 107 | 38.2 | 50 | 17.9 | 57 | 20.4 |  |  | 85 | 30.4 | 22 | 7.9 |  |  |
| **Have had previous surgery** |  |  |  |  |  |  |  |  |  |  |  |  |  |  |
| No | 86 | 30.7 | 36 | 12.9 | 50 | 17.9 | 3.3 | 0.092 | 69 | 24.6 | 17 | 6.1 | 0.0 | 1.000 |
| Yes | 194 | 69.3 | 104 | 37.1 | 90 | 32.1 |  |  | 155 | 55.4 | 39 | 13.9 |  |  |
| **Have had surgical complications** | |  |  |  |  |  |  |  |  |  |  |  |  |  |
| No | 253 | 90.4 | 130 | 46.4 | 123 | 43.9 | 2.0 | 0.224 | 205 | 73.2 | 48 | 17.1 | 1.7 | 0.207 |
| Yes | 27 | 9.6 | 10 | 3.6 | 17 | 6.1 |  |  | 19 | 6.8 | 8 | 2.9 |  |  |
| **Type of surgery** |  |  |  |  |  |  |  |  |  |  |  |  |  |  |
| General | 84 | 30.0 | 46 | 28.0 | 38 | 28.0 | 3.5 | 0.063 | 66 | 28.0 | 18 | 28.0 | 14.3 | 0.055 |
| Obstetrics and gynecology | 63 | 22.5 | 14 | 28.0 | 49 | 28.0 |  |  | 42 | 28.0 | 21 | 28.0 |  |  |
| Orthopedic | 44 | 15.7 | 30 | 28.0 | 14 | 28.0 |  |  | 39 | 28.0 | 5 | 28.0 |  |  |
| Ear, nose, and throat | 22 | 7.9 | 9 | 28.0 | 13 | 28.0 |  |  | 18 | 28.0 | 4 | 28.0 |  |  |
| Urology | 25 | 8.9 | 14 | 28.0 | 11 | 28.0 |  |  | 22 | 28.0 | 3 | 28.0 |  |  |
| Ophthalmology | 4 | 1.4 | 2 | 28.0 | 2 | 28.0 |  |  | 3 | 28.0 | 1 | 28.0 |  |  |
| Neurosurgery | 15 | 5.4 | 12 | 28.0 | 3 | 28.0 |  |  | 15 | 28.0 | 0 | 28.0 |  |  |
| Cardiac surgery/intervention | 15 | 5.4 | 9 | 28.0 | 6 | 28.0 |  |  | 13 | 28.0 | 2 | 28.0 |  |  |
| Minor surgeries/interventions | 8 | 2.9 | 4 | 28.0 | 4 | 28.0 |  |  | 6 | 28.0 | 2 | 28.0 |  |  |
